# Identification of Novel *GCK* and *HNF4α* Gene Variants in Japanese Pediatric Patients with Onset of Diabetes before 17 Years of Age

**DOI:** 10.1155/2021/7216339

**Published:** 2021-10-29

**Authors:** Rumi Katashima, Mari Matsumoto, Yuka Watanabe, Maki Moritani, Ichiro Yokota

**Affiliations:** ^1^Laboratory for Pediatric Genome Medicine, Department of Clinical Research, National Hospital Organization Shikoku Medical Center for Children and Adults, 2-1-1 Senyu-cho, Zentsuji City, Kagawa 765-8507, Japan; ^2^Department of Endocrinology and Metabolism, Faculty of Medicine, Kagawa University, 1750-1, Miki-cho, Kita-gun, Kagawa 761-0793, Japan; ^3^Department of Pediatric Endocrinology and Metabolism, National Hospital Organization Shikoku Medical Center for Children and Adults, 2-1-1, Senyu-cho, Zentsuji City, Kagawa 765-8507, Japan

## Abstract

**Background:**

Maturity-onset diabetes of the young (MODY) is commonly misdiagnosed as type 1 or type 2 diabetes. Common reasons for misdiagnosis are related to limitations in genetic testing. A precise molecular diagnosis is essential for the optimal treatment of patients and allows for early diagnosis of their asymptomatic family members.

**Objective:**

The aim of this study was to identify rare monogenic variants of common MODY genes in Japanese pediatric patients.

**Methods:**

We investigated 45 Japanese pediatric patients based on the following clinical criteria: development of diabetes before 17 years of age, a family history of diabetes, testing negative for glutamate decarboxylase-65 (GAD 65) antibodies and insulinoma-2-associated autoantibodies (IA-2A), no significant obesity, and evidence of endogenous insulin production. Genetic screening for MODY1 (*HNF4α*), MODY2 (*GCK*), MODY3 (*HNF1α*), and MODY5 (*HNF1β*) was performed by direct sequencing followed by multiplex ligation amplification assays.

**Results:**

We identified 22 missense variants (3 novel variants) in 27 patients (60.0%) in the *GCK*, *HNF4α*, and *HNF1α* genes. We also detected a whole exon deletion in the *HNF1β* gene and an exon 5–6 aberration in the *GCK* gene, each in one proband (4.4%). There were a total of 29 variations (64.4%), giving a relative frequency of 53.3% (24/45) for *GCK*, 2.2% (1/45) for *HNF4α*, 6.7% (3/45) for *HNF1α*, and 2.2% (1/45) for *HNF1β* genes.

**Conclusions:**

Clinicians should consider collecting and assessing detailed clinical information, especially regarding *GCK* gene variants, in young antibody-negative patients with diabetes. Correct molecular diagnosis of MODY better predicts the clinical course of diabetes and facilitates individualized management.

## 1. Introduction

Maturity-onset diabetes of the young (MODY) (MIM 606391) is traditionally characterized by early onset (before 25 years of age) and a heterogeneous autosomal dominant form of inheritance. It accounts for 1% to 6.5% of pediatric diabetes cases in Europe in nationwide population-based studies [1–4]. However, the prevalence in Japanese pediatric patients is not clear, because the diagnostic rates have primarily been low in studies of adult patients [5].

MODY is often misdiagnosed as type 1 (T1D) or type 2 diabetes (T2D) because of the overlap of clinical characteristics [6, 7]. Patients are, therefore, often not offered an optimal treatment regimen [8]. Misdiagnosis of MODY arises because the phenotype of monogenic diabetes is not sufficiently distinctive to allow for easy clinical differentiation of patients with MODY from those with the more common forms of diabetes. Thus, the molecular genetic diagnosis of MODY is important for clinical decisions, treatment, and genetic counseling [9–11]. Currently, however, the rate of genetic diagnosis of monogenic diabetes is low because of the limited phenotype information available and the high costs of genetic testing. These are the typical challenges involved in differentiating patients, and a molecular genetic diagnosis can help identify patients with MODY.

Based on linkage analysis, MODY is defined by variants in different genes, of which at least 13 genes are known on OMIM. Almost all MODY cases are attributable to highly penetrant variants in the four most common MODY genes, namely, MODY1, 2, 3, and 5 (specifically hepatocyte nuclear factor 4-alpha (*HNF4α*; MODY1), glucokinase (*GCK*; MODY2), hepatocyte nuclear factor 1-alpha (*HNF1α*; MODY3), and hepatocyte nuclear factor 1-beta (*HNF1β*; MODY5)) in Caucasians [12]. Studies on Japanese pediatric patients with MODY have shown a variable prevalence of the different MODY subtypes. In Japan, MODY1, 2, 3, and 5 are the most common forms, while MODY4 and 6 are rare. However, there may be patients with MODY that have not been identified, especially with MODY2.

In this study, we focused on the four most common MODY genes (*HNF4α*, *GCK*, *HNF1α*, and *HNF1β*) and performed screening analysis of 45 Japanese pediatric patients who were between 2.8 and 17 years of age at diagnosis. Each genetic subtype of MODY demands a different management and treatment strategy. Thus, genetic diagnosis is the key to adequate treatment and requires proper interpretation and assessment of both known and novel variants.

## 2. Materials and Methods

### 2.1. Subjects and Clinical Studies

We recruited 45 (17 males and 28 females) unrelated Japanese probands with diabetes clinically characterized as MODY. Members of each family participated. The probands with diabetes were enrolled across 14 unrelated hospitals between 2009 and 2017 in Japan.

Probands were between 2.8 and 17 years of age at the time of diagnosis. In this study, we selected the MODY subjects for genetic testing, based on the following clinical criteria: (1) onset of diabetes before 25 years of age, (2) a family history of diabetes, (3) testing negative for glutamate decarboxylase-65 (GAD 65) antibodies and insulinoma-2-associated autoantibodies (IA-2A), (4) no significant obesity, and (5) evidence of endogenous insulin production.

The clinical diagnosis of MODY was based on the clinical features, laboratory records, and the guidelines of the Expert Committee Report of the American Diabetes Association. Hemoglobin (Hb) A1c levels were measured according to the National Glycohemoglobin Standardization Program (NGSP) [13] using the International System of Units (SI) [14]. The clinical characteristics of the enrolled patients are provided in Table 1.

Control samples (*n* = 100) were mainly recruited from the Health Science Research Resources Bank of the Japanese Collection of Research. These were members of the general population who underwent medical checkups to confirm that they had no family history of diabetes. Genomic DNA was extracted from the subjects with diabetes using a standard protocol.

The study was approved by the Ethics Committee of the Shikoku Medical Center for Children and Adults (Kagawa, Japan) (approval No. H20-31). This study was conducted in accordance with the principles of the Declaration of Helsinki. Written informed consent was obtained from the parents/guardians of the children and/or the subjects themselves.

### 2.2. Identification of MODY Genes

The most common forms of MODY are MODY1–3 and MODY5, while MODY4 and MODY6 are rare in the Japanese population (6). Thus, we performed mutational screening for the four relevant genes in MODY1, MODY2, MODY3, and MODY5 (*HNF4α* (gene ID: 3172), *GCK* (gene ID: 2645), *HNF1α* (gene ID: 6927), and *HNF1β* (gene ID: 6928), respectively) by a direct sequencing method, as described in our previous report [15]. The primers used are listed in Supplemental Table 1. The three novel variants were identified by comparison with the results of 100 control subjects.

All sequences were compared with the following GenBank reference sequences using GENETYX®Win v8.0 (Genetyx, Tokyo, Japan): NM_175914.3, NM_000457.3, and NP_787110.2 for *HNF4α*; NM_000162.3 and NP_000153.1 for *GCK*; NM_000545.5 and NP_000536.5 for *HNF1α*; and NM_000458.2 and NP_000449.1 for *HNF1β*.

### 2.3. In Silico Analysis for Novel Variants

Possible functional effects of the identified variants, especially the unknown variants, were identified with two web-based programs, PolyPhen 2 and SIFT. Potential effects on splicing were evaluated with HSF (Human Splicing Finder). Clinical information was evaluated with HGMD Professional, ClinVar, and ExAC. Sequence conservation was evaluated with HGMD Professional.

### 2.4. Multiplex Ligation Amplification (MLPA) Assay

We examined DNA copy number variations in *HNF4α*, *GCK*, *HNF1α*, and *HNF1β* genes in the probands in whom definite mutations were not identified by sequencing. The MLPA reactions were performed according to the manufacturer's general instructions using SALSA MLPA Probemix P241-B1 MODY Mix1 (MRC-Holland, Amsterdam, Netherlands) and were analyzed on a 3130 Genetic Analyzer (Applied Biosystems). DNA from three human genome control samples, namely, male, female, and mixed (male + female) (Promega, WI, USA), was used as reference DNA.

### 2.5. Custom Array Comparative Genomic Hybridization (CGH) Assay

To confirm copy number aberrations, we also performed a custom CGH microarray specialized for the *HNF1β* gene using the Agilent array database ver. 6.5 (http://earray.chem.agilent.com/array/; Agilent Technologies, CA, USA). The high-resolution array CGH was used to design a probe set covering the Chr17 genomic region (31,800,001–38,100,000) mapping around the *HNF1β* gene. This probe set included 31,500 probes on 17q12 (average spacing = 200 bp) and was duplicated to confirm the results and attain the 1 × 244K array format. The protocols used for probe labeling, hybridization, and data analysis were described by Cell Innovator Corp.

## 3. Results

### 3.1. Patient Characteristics

The clinical characteristics of the 45 probands are summarized in Table 1. All probands were diagnosed before 20 years of age; the average age was 10.5 ± 3.66 years at the time of diagnosis. The incidence of a family history of diabetes was 100%, and 73.3% (33/45) spanned three generations. HbA1c analysis and oral glucose tolerance tests (OGTT) in probands revealed hyperglycemia during fasting (at OGTT 0 min) without ketoacidosis (except in one proband). The diagnosis of hyperglycemia in most probands was either fortuitous or made using the screening system based on glycosuria in schools.

### 3.2. Genetic Variants in MODY Genes

**Figure 1:**
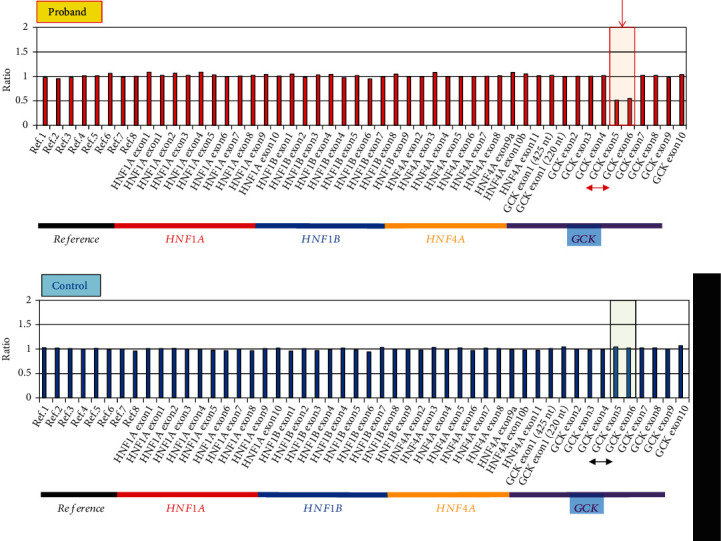
Detection of the exon 5–6 hemizygous deletion in the *GCK* gene in leukocyte genomic DNA by MLPA assay. Graphical representation of the *GCK* gene. Chromosome X and Y probes were normalized to the three control samples in the proband (a) and control (b). *GCK* probes in exons 5 and 6 are expressed in half dosages in the proband, indicating the deletion of exon 5–6. The exon 5–6 deletion is marked with a red arrow and square.

**Figure 2:**
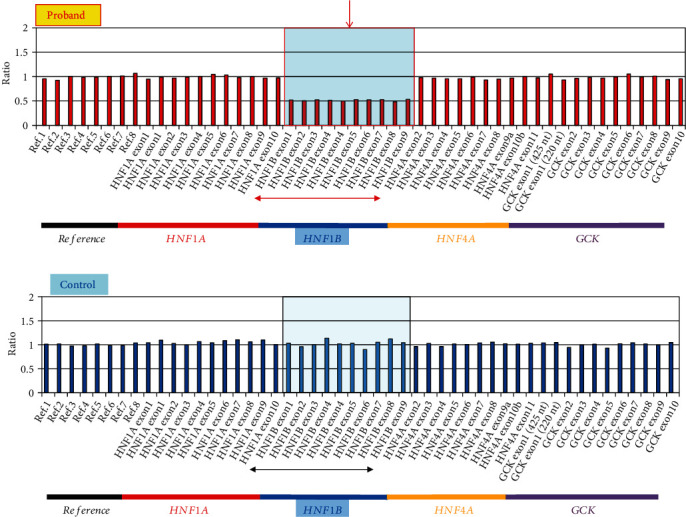
Detection of a whole hemizygous deletion in the *HNF1β* gene in leukocyte genomic DNA by MLPA assay. Graphical representation of the *HNF1β* gene. Chromosome X and Y probes were normalized to the control in the proband (a) and control (b). MLPA results show that all *HNF1β* probes are expressed in half dosages in the proband relative to the expected heights indicating a whole-gene hemizygous deletion change in *HNF1β*. The whole-gene deletion of *HNF1β* is marked with a red arrow and square.

We identified a total of 22 missense variants (3 novel variants) in 27 patients (60.0%) in the *GCK*, *HNF4α*, and *HNF1α* genes in this study involving a small set of 45 Japanese pediatric MODY patients (Table 2). Additionally, we detected an exon 5–6 deletion in the *GCK* gene (Figure 1) and a whole coding exon deletion in the *HNF1β* gene (Figure 2) in one proband each (2.2% each). There were a total of 29 probands (64.4%), giving a relative frequency of 53.3% (24/45) for *GCK*, 2.2% (1/45) for *HNF4α*, 6.7% (3/45) for *HNF1α*, and 2.2% (1/45) for *HNF1β* genes.

#### 3.2.1. Screening for the GCK Gene

We identified 18 missense variants (2 novel and 16 previously described variants [16–27] in 23 probands (Table 2). Most of these variants were found in one proband, while G223S and R377S were found in two probands and T228M was identified in four probands. Of these missense changes, two (G147D and N254D) were novel (Supplemental Figure 1_A, B), and all changes were heterozygous. Neither of the novel missense changes (G147D and N254D) were identified in the 100 healthy controls, suggesting that these are diabetes-specific variants. The 18 variant sites bearing the missense variants were conserved across species (data not shown).

We identified an exon 5–6 hemizygous deletion in the *GCK* gene in leukocyte genomic DNA from one proband (Figure 1). The *GCK* gene variants had the highest frequency among the MODY gene variants.

#### 3.2.2. Screening for the HNF4*α* Gene

We identified one missense heterozygous variant in the *HNF4α* gene. This missense variant, V251G (exon 7), was novel (Supplemental Figure 1_C) and found in one proband. This novel missense change was not identified in the healthy controls, suggesting that it is a diabetes-specific variant.

#### 3.2.3. Screening for the HNF1*α* Gene

We identified three known missense variants in the *HNF1α* gene [24, 28, 29]. These changes, A116V, R131W, and R271W, which occurred in one proband each, were all heterozygous. The other missense changes identified (I27L (exon 1) and S487N (exon 7); data not shown) were also detected in healthy controls (I27L: 52% (heterozygous) and 23% (homozygous); S487N: 59% (heterozygous) and 22% (homozygous) in controls), suggesting that they are polymorphism changes.

#### 3.2.4. Screening for the HNF1*β* Gene

No missense changes were identified in the *HNF1β* gene. However, we identified a known whole-gene hemizygous deletion in *HNF1β* in leukocyte genomic DNA from one proband by MLPA, as peaks for whole exons in *HNF1β* were half the expected height [30] (Figures 2(a) and 2(b)). Unfortunately, we could not obtain DNA from parents or other family members of this proband at the time of this study. Therefore, we were unable to screen for this change in relatives of this proband.

To confirm this observation and assess the breakpoints of hemizygous deletion in *HNF1β*, we performed whole-genome array CGH. We confirmed a 1.7-Mb hemizygous deletion on chromosome 17q12 (Supplemental Figure 2). A magnified view of the array CGH indicated that the genomic deletion included the *HNF1β* gene and 15 other genes (*LHX1*, *ZNHIT3*, *MYO19*, *PIGW*, *GGNBP2*, *DHRS11*, *MRM1*, *AATF*, *ACACA*, *C17orf78*, *TADA2A*, *DUSP14*, *SYNRG*, *DDX52*, and *LOC284100*).

### 3.3. Family History Analysis

In this study, although DNA of all of the probands' parents was not available for genetic testing, the family histories of all probands were based on genetic counseling information. Among the 29 probands with variants (*GCK* = 24, *HNF*1*α* = 3, *HNF*4*α* = 1, and *HNF*1*β* = 1), parental DNA was available for only 17 probands (Supplemental Figure 3).

Among these 17 probands, DNA from at least three generations was available for only one family. All families had a heterozygous variant consistent with that of their child in 2 consecutive generations. In one family with the L324P variant in the *GCK* gene, the proband's mother had normal alleles, while DNA from one parent (F-42_I.1) was not available. Therefore, care should be taken, as that parent may also have MODY variants.

### 3.4. Clinical Characteristics of Patients with Variants in Four Genes and MODY X (with Unknown Genetic Etiology)

Based on the screening results, the clinical characteristics of the affected individuals with *GCK*, *HNF1α*, and *HNF4α* variants are presented in Supplemental Table 2. The age at diagnosis of diabetes was higher in *HNF4α*- and *HNF1β*-MODY probands than in the other MODY types, including MODY X. MODY X probands exhibited higher fasting glucose levels and glucose levels at 60 min during an OGTT compared with those of *GCK*-, *HNF1α*-, and *HNF4α*-MODY probands. Insulin secretion, as assessed by the 60 min insulin-to-glucose ratio during an OGTT, was lower in *HNF4α*-MODY patients than in other MODY probands, indicating the presence of a more severe glucose-stimulated insulin secretory defect, although the comparison was made against only one subject. Further studies that include other *HNF4α*-MODY probands are required to completely rule this result out. In contrast, *GCK*-MODY patients displayed increased insulin secretion at 60 min during the OGTT relative to the levels in the other MODY groups. The prevalence of obesity and HbA1c was not different between groups.

The sample size for this study was small. Our mutational analysis of only *GCK*, *HNF4α*, *HNF1α*, and *HNF1β* genes is not sufficient to strictly confirm the susceptibility mutation in patients with an onset of diabetes at an early age. Due to the small number of MODY cases, which represent only a small percentage of pediatric diabetes cases, and the high costs of genetic analysis, it is not easy to increase the sample size and include other candidate genes for analysis.

## 4. Discussion

Correct genetic diagnosis of MODY is important because it better predicts the clinical course of diabetes and facilitates individualized management. In this study, 22 missense variants in the *GCK*, *HNF4α*, and *HNF1α* genes and 2 deletions in the *GCK* and *HNF1β* genes were identified in 29 probands (64.5%). These included three novel variants affecting the amino acid residues G147D and N254D in *GCK* and V251G in *HNF4α*. Although these variants occurred rarely and were each found in only one individual, none of these variants were found in control patients. Based on these observations and predicted functional effects from in silico analysis, we concluded that these variants are putatively associated with MODY phenotypes in Japanese pediatric patients.

*GCK*-MODY causes a mild, asymptomatic diabetes and stable fasting hyperglycemia usually requiring no specific treatment. Generally, the incidence of *GCK* variants has been reported less frequently than other MODY types in Japanese patients [20]. However, the incidence is mainly based on studies on adult subjects, and this result is similar to those of reports from other Asian populations [31–33]. In patients with pediatric onset T2D that are young and nonobese and have a family history of MODY or persistent mild fasting hyperglycemia, there may be a genetic variant in the *GCK* gene [6, 34]. Thus, we hypothesized that *GCK* gene variants would be common in nonobese patients without autoantibodies with asymptomatic diabetes in school medical examinations.

Indeed, according to a systematic approach using detailed laboratory data and family history, we identified 19 *GCK* variants in 24 probands. This frequency was higher than that of other MODY genes. A *GCK* variant should be considered if there is significant family history. Most *GCK* gene variants are generally single nucleotide variants, and *GCK* deletions are rare [35]. In this study, we identified one small deletion encompassing exon 5–6 of the *GCK* gene in one proband (F-17 in Supplemental Figure 3). This deletion was also identified in this proband's father, indicating cosegregation with the onset of diabetes.

In Japan, variants in the *HNF1α* gene are the most commonly identified MODY genes, which is consistent with studies on the European population [5]. Variants in the *HNF1α/HNF4α* genes display very similar clinical characteristics. The most striking features of *HNF1α*-/*HNF4α-*MODY are that they result in a reduced insulin secretory response to glucose but marked sensitivity to low-dose sulfonylureas [36] and cause progressive pancreatic *β*-cell dysfunction and increasing hyperglycemia that leads to diabetes in early adult life. These features are similar our patients with *HNF1α*-/*HNF4α-*MODY. The patient with the *HNF4α* variant revealed a novel heterozygous variation at V251G (exon 7). Unfortunately, we were unable to obtain DNA from the parents or sibling of this proband at the time of this study.

*HNF1β-*MODY is characterized by renal dysfunction, including renal cysts, renal dysplasia, pancreatic atrophy, and genital abnormalities [37]. We identified a hemizygous whole-gene deletion in the *HNF1β* gene in a 17-year-old girl with nephropathy, renal cysts in the left kidney, and absence of a right kidney as identified by computed tomography scans, along with loss of the vagina after following the clinical characteristics of high glucose levels and impaired insulin secretion. The results of genetic testing were consistent with our speculation about the abnormality in the *HNF1β* gene. Our data suggests that according for the *HNF1β* variant, the typical phenotype is more important than the family history, because this case was detected without family analysis.

Generally, approximately 60% or more of clinically diagnosed MODY cases can be attributed to a single gene. However, the remaining 20% to 30% of MODY cases are likely due to an unidentified gene, as in MODY-X cases in Caucasians [38], and this percentage is even higher in Asian (including Japanese) populations [39]. In Japanese populations, MODY-X may account for 20–50% of patients with MODY. Recently, a clinical prediction model has been developed to predict the probability of MODY in young patients [40]. Tools such as the MODY calculator could be of significant assistance in considering a MODY diagnosis in the first steps. This tool is available at http://www.diabetesgenes.org/content/mody-probability-calculator. Rapid genetic MODY diagnosis is important, but diagnosing MODY subtypes is difficult for physicians, and most patients with MODY remain undiagnosed. In current diagnostic testing, next-generation sequencing (NGS) technology provides the potential for simultaneous analysis of the known disease genes in a single assay [31, 41]. We are now planning to develop a targeted NGS assay to identify variants causing monogenic diabetes for patients with MODY without known variants.

The results of this study suggested that improved clinical specificity can allow for better detection of MODY variants. Interestingly, we identified 20 patients (83.3%) with variants in the *GCK* gene (all but 4 cases) or *HNF1β* deletion (100%) by single target gene analysis. These patients with variants can be associated with diabetes phenotypes or clinical features involving the *GCK* or *HNF1β* gene, such as age of onset of diagnosis, level of secretion of insulin during an OGTT, or renal dysplasia or abnormality. In contrast, it was difficult to identify the variant for probands who could not be diagnosed with a specific MODY subtype due to limited phenotype information. We were only able to identify the variant against a single gene in one case (5.0%, 1/20). Thus, there are close genotype-phenotype correlations between the genetic variant and the clinical features. We strongly suggest that “genotype-phenotype-specific candidate gene selection” is important in order to reduce the unidentified variants, and screening will become more cost-effective with advances in research.

## 5. Conclusion

A molecular genetic diagnosis of disease-causing genes allows for more appropriate management and can help identify affected and at-risk family members. Diagnosing MODY is a challenge for physicians, and the majority of cases remain unidentified. A nationwide and systematic approach is required for the rapid diagnosis and appropriate management of MODY.

## Figures and Tables

**Table 1:** Subject characteristics (*n* = 45).

Phenotype	Subjects
Male/female	17/28^a^
Age (years) at the time of diagnosis	10.5 ± 3.66
Age at diabetes onset	
≤5 years	5/1^a^
>5 years to ≤10 years	4/10
>10 years to ≤15 years	8/15
>15 years to ≤25 years	0/2
Blood glucose concentrations (mg/dL)	
OGTT 0 min	131.8 ± 31.31
OGTT 60 min	227.5 ± 62.04
OGTT 120 min	205.7 ± 56.32
IRI (*μ*U/mL)	
OGTT 0 min	5.5 ± 3.21
OGTT 60 min	37.7 ± 26.95
OGTT 120 min	50.4 ± 33.95
HbA1c (%) at diagnosis (NGSP)	7.53 ± 1.69
mmol/mol (SI)	59
^b^Body mass index (kg/m^2^)/obesity index (%)	18.1 ± 2.73/−0.55 ± 0.19
C-peptide (ng/mL) (pre)	1.8 ± 1.20
*β*-Cell antibodies (GAD/IA-2A)	No
Diabetic ketoacidosis	1/45
Urine glucose	39/46 (86.7%)
^c^Family history of diabetes (%)	
In one parent	40/45 (88.9%)
In a second-degree relative	33/45 (73.3%)
In a third-degree relative	14/45 (31.1%)
In a sibling	4/456 (8.9%)

Age at the time of diagnosis, blood glucose concentrations, HbA1c, and insulin dose are represented as means ± standard deviation. ^a^Left/right denotes male/female. ^b^All subjects were non-obese. ^c^Number of subjects with a sibling, or first–third-degree relative with type 1 or type 2 diabetes. NGSP: National Glycohemoglobin Standardization Program; SI: International System of Units; ND: no data.

**Table 2:** Missense variants and exon deletion of MODY genes in Japanese probands.

Gene	Protein variant^a^	Location	Nucleotide changes	Carrier frequency			Clinical information	Possible functional impact			Conservation (protein)	Class
				Patients	Controls (*n* = 100)	Refs.	HGMD professional	Protein level	RNA level			
								Polyphen-2/SIFT	Splice	ESE/ESS		
*GCK*	M41T	Exon 2	c.122 T>C	1	0	[16]	DM	Possibly damaging/deleterious	No	Possible/no	Yes	Pathogenic
	T60I	Exon 2	c.179 C>T	1	NA	[17]	DM	Possibly damaging/deleterious	No	Possible/no	Yes	Pathogenic
	G72R	Exon 3	c.214 G>A	1	NA	[18]	DM	Possibly damaging/deleterious	Yes	No/possible	Yes	Pathogenic
	L77P	Exon 3	c.230 T>C	1	NA	[19]	DM	Possibly damaging/deleterious	No	Possible/no	Yes	Pathogenic
	G81C	Exon 3	c.241 G>T	1	0	[20]	DM	Possibly damaging/deleterious	No	Possible/no	Yes	Pathogenic
	**G147D**	Exon 4	c.440 G>A	1	0	Unknown	—	Possibly damaging/deleterious	No	No/no	—	Unknown significant
	T206M	Exon 6	c.617 C>T	1	NA	[21]	DM	Possibly damaging/deleterious	No	No/possible	Yes	Pathogenic
	G223S	Exon 6	c.667 G>A	2	0	[21–22]	DM	Possibly damaging/deleterious	No	Possible/no	Yes	Pathogenic
	T228M	Exon 7	c.683 C>T	4	0	[23]	DM	Possibly damaging/deleterious	No	Possible/possible	Yes	Pathogenic
	Q239R	Exon 7	c.716 A>G	1	0	[24]	DM	Benign/tolerated	No	Possible/no	No	Likely pathogenic
	**N254D**	Exon 7	c.760 A>G	1	0	Unknown	—	Possibly damaging/deleterious	No	Possible/no	—	Unknown significant
	L324P	Exon 8	c.971 T>C	1	NA	[25]	DM	Possibly damaging/deleterious	No	Possible/no	Yes	Likely pathogenic
	S336X	Exon 8	c.1,007 C>A	1	0	[16–20]	DM	Possibly damaging/deleterious	No	Possible/possible	—	Pathogenic
	R377H	Exon 9	c.1,130 G>A	1	0	[19]	DM	Possibly damaging/deleterious	No	Possible/no	Yes	Likely pathogenic
	R377S	Exon 9	c.1,129 C>A	2	NA	[16]	DM	Possibly damaging/deleterious	Yes	No/possible	Yes	Pathogenic
	S383L	Exon 9	c.1,148 C>T	1	NA	[26]	DM	Possibly damaging/deleterious	No	No/possible	No	Pathogenic
	R392C	Exon 9	c.1,174 C>T	1	0	[27]	DM	Possibly damaging/deleterious	No	Possible/possible	No	Likely pathogenic
	A454V	Exon 10	c.1,361 C>T	1	NA	[27]	DM	Possibly damaging/deleterious	Yes	No/possible	Yes	Pathogenic
	E5-6 deletion	Exon 5-6	—	1	NA	[16]	DM	—	—	—	—	Pathogenic

*HNF4α*	**V251G**	Exon 7	c.752 T>G	1	0	Unknown	—	Possibly damaging/deleterious	Yes	Possible/no	—	Unknown significant

*HNF1α*	A116V	Exon 2	c.347 C>T	1	NA	[24]	DM	Possibly damaging/deleterious	Yes	Possible/possible	Yes	Pathogenic
	R131W	Exon 2	c.391 C>T	1	0	[28]	DM	Possibly damaging/deleterious	Yes	No/possible	Yes	Pathogenic
	R271W	Exon 4	c.811 C>T	1	NA	[29]	DM	Possibly damaging/deleterious	No	Possible/no	Yes	Pathogenic

*HNF1β*	Whole gene deletion		—	1	NA	[30]	DM	—	—	—	—	Pathogenic

Amino acid numbers and nucleotide changes are based on NCBI RefSeq NM_175914.3 and NP_787110.2 for HNF4*α*, NM_000162.3 and NP_000153.1 for GCK, and NM_000545.5 and NP_000536.5 for HNF1*α*. ^a^Bold font denotes novel missense mutations in diabetes-specific missense mutations. All changes were heterozygous. NA: not analyzed. Possible functional effects of identified variants, especially unknown (novel) variants, were identified with two web-based programs, PolyPhen 2 (http://genetics.bwh.harvard.edu/pph2/) and SIFT (http://sift.bii.a-star.edu.sg/www/SIFT_seq_submit2.html). Potential effects on splicing were evaluated with HSF (Human Splicing Finder; http://www.umd.be/HSF3/technicaltips.html). Clinical information was evaluated with HGMD professional (http://www.hgmd.cf.ac.uk/) and ClinVar (http://www.ncbi.nlm.nih.gov/clinvar/). Sequence conservation was evaluated with HGMD professional (http://www.hgmd.cf.ac.uk/).

## Data Availability

The confidential data used to support the findings of this study are restricted by the ethics committee in order to protect patient privacy.

## Abbreviations

MODY: Maturity-onset diabetes of the young
MODY1: Hepatocyte nuclear factor 4-alpha (*HNF4α*)
MODY2: Glucokinase (*GCK*)
MODY3: Hepatocyte nuclear factor 1-alpha (*HNF1α*)
MODY5: Hepatocyte nuclear factor 1-beta (*HNF1β*)
T1D: Type 1 diabetes
T2D: Type 2 diabetes
MLPA: Multiplex ligation amplification
CGH: Comparative genomic hybridization
OGTT: Oral glucose tolerance tests
NGS: Next-generation sequencing.

## Supplementary Materials

Supplementary Materials Supplemental Table 1: primers used for *HNF4α*, *GCK*, *HNF1α*, and *HNF1β* genes for sequencing analysis. Supplemental Table 2: clinical characteristics of MODY cases with or without variants in each gene. Supplemental Figure 1: sequences of two novel *GCK* gene mutations and one novel *HNF4α* gene mutation. Supplemental Figure 2: results of array CGH (human genome CGH array 244K). Supplemental Figure 3: pedigrees of the families of the probands with mutations in *GCK*, *HNF1α*, *HNF4α*, and *HNF1β* genes, respectively.
