# Supplementary material for: Identification of Novel GCK and HNF4α Gene Variants in Japanese Pediatric Patients with Onset of Diabetes before 17 Years of Age
Source: J Diabetes Res. 2021 Oct 29;2021:7216339. doi: 10.1155/2021/7216339 (PMC8570896; doi:10.1155/2021/7216339)
Supplement: Supplementary Materials — Supplemental Table 1: primers used for HNF4α, GCK, HNF1α, and HNF1β genes for sequencing analysis. Supplemental Table 2: clinical characteristics of MODY cases with or without variants in each gene. Supplemental Figure 1: sequences of two novel GCK gene mutations and one novel HNF4α gene mutation. Supplemental Figure 2: results of array CGH (human genome CGH array 244K). Supplemental Figure 3: pedigrees of the families of the probands with mutations in GCK, HNF1α, HNF4α, and HNF1β genes, respectively. [file 7216339.f1.zip › Supplemental Figure 3.pptx]

## Slide 1
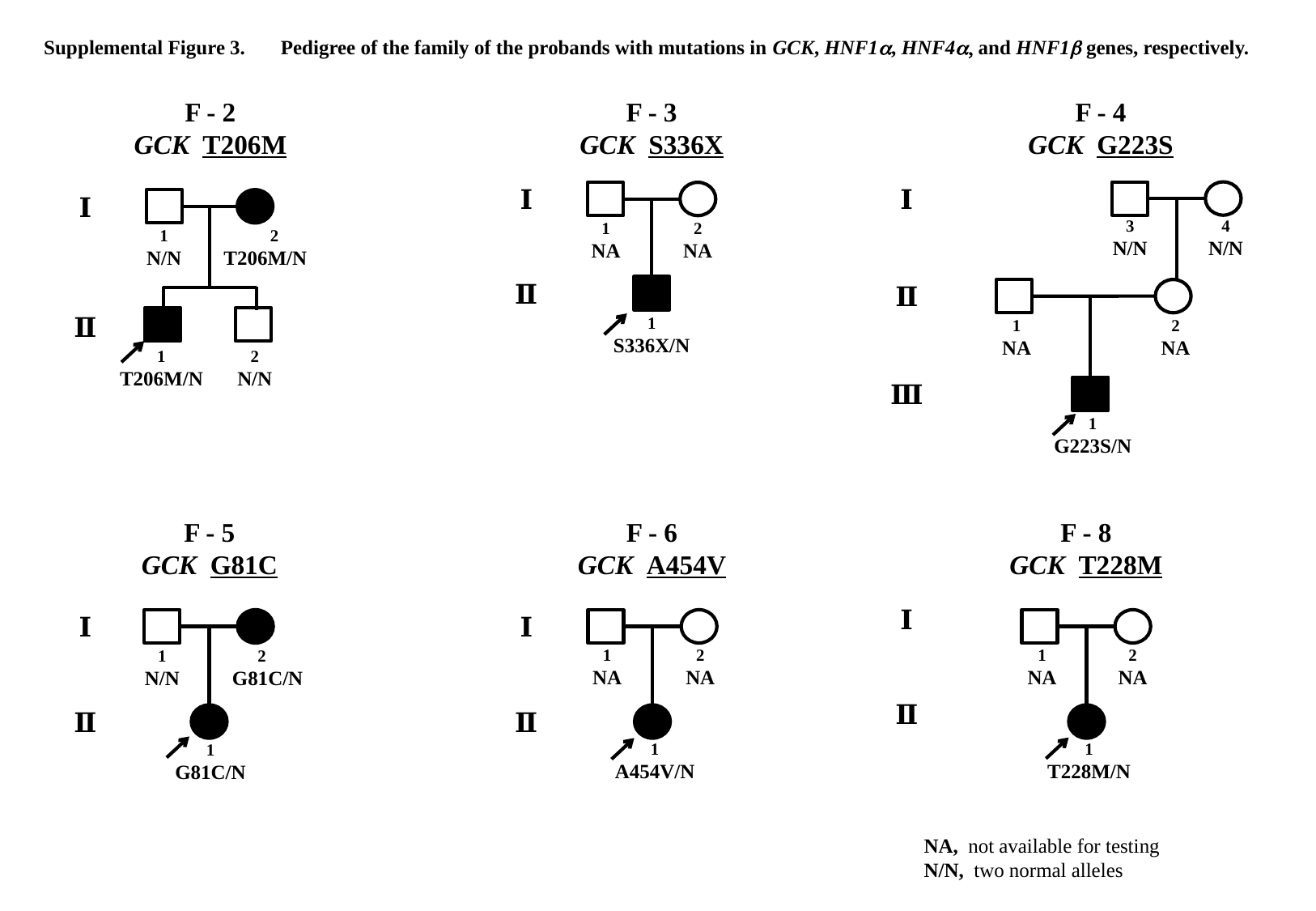

Supplemental Figure 3.　 Pedigree of the family of the probands with mutations in GCK, HNF1a, HNF4a, and HNF1b genes, respectively.
F - 2
GCK T206M
Ⅰ
1
N/N
　　 2
T206M/N
Ⅱ
1
T206M/N
2
N/N
F - 3
GCK S336X
Ⅰ
1
NA
2
NA
Ⅱ
1
S336X/N
F - 4
GCK G223S
Ⅰ
3
N/N
4
N/N
Ⅱ
1
NA
2
NA
Ⅲ
1
G223S/N
F - 5
GCK G81C
Ⅰ
1
N/N
 2
G81C/N
Ⅱ
1
G81C/N
F - 6
GCK A454V
Ⅰ
1
NA
2
NA
Ⅱ
1
A454V/N
F - 8
GCK T228M
2
NA
1
T228M/N
1
NA
Ⅰ
Ⅱ
NA, not available for testing
N/N, two normal alleles

## Slide 2
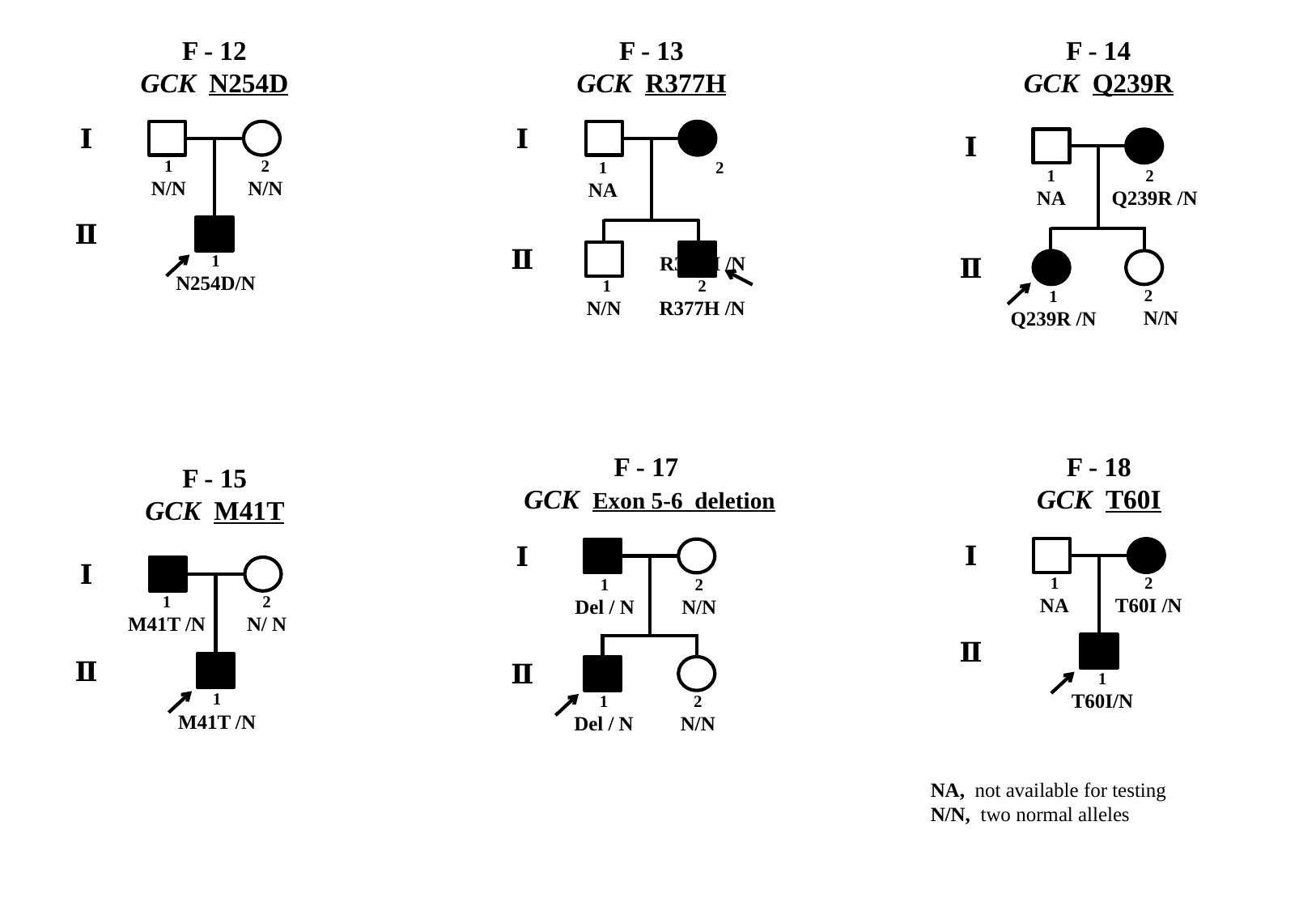

F - 12
GCK N254D
Ⅰ
1
N/N
2
N/N
Ⅱ
1
N254D/N
F - 13
GCK R377H
Ⅰ
　　 2　　　　　　　　　　　R377H /N
1
NA
Ⅱ
 1
 N/N
2
R377H /N
F - 14
GCK Q239R
Ⅰ
1
NA
 2
Q239R /N
Ⅱ
2
　N/N
1
Q239R /N
F - 17
GCK Exon 5-6 deletion
Ⅰ
1
Del / N
2
N/N
Ⅱ
1
Del / N
2
N/N
F - 18
GCK T60I
Ⅰ
1
NA
2
T60I /N
Ⅱ
1
T60I/N
F - 15
GCK M41T
Ⅰ
1
M41T /N
2
N/ N
Ⅱ
1
M41T /N
NA, not available for testing
N/N, two normal alleles

## Slide 3
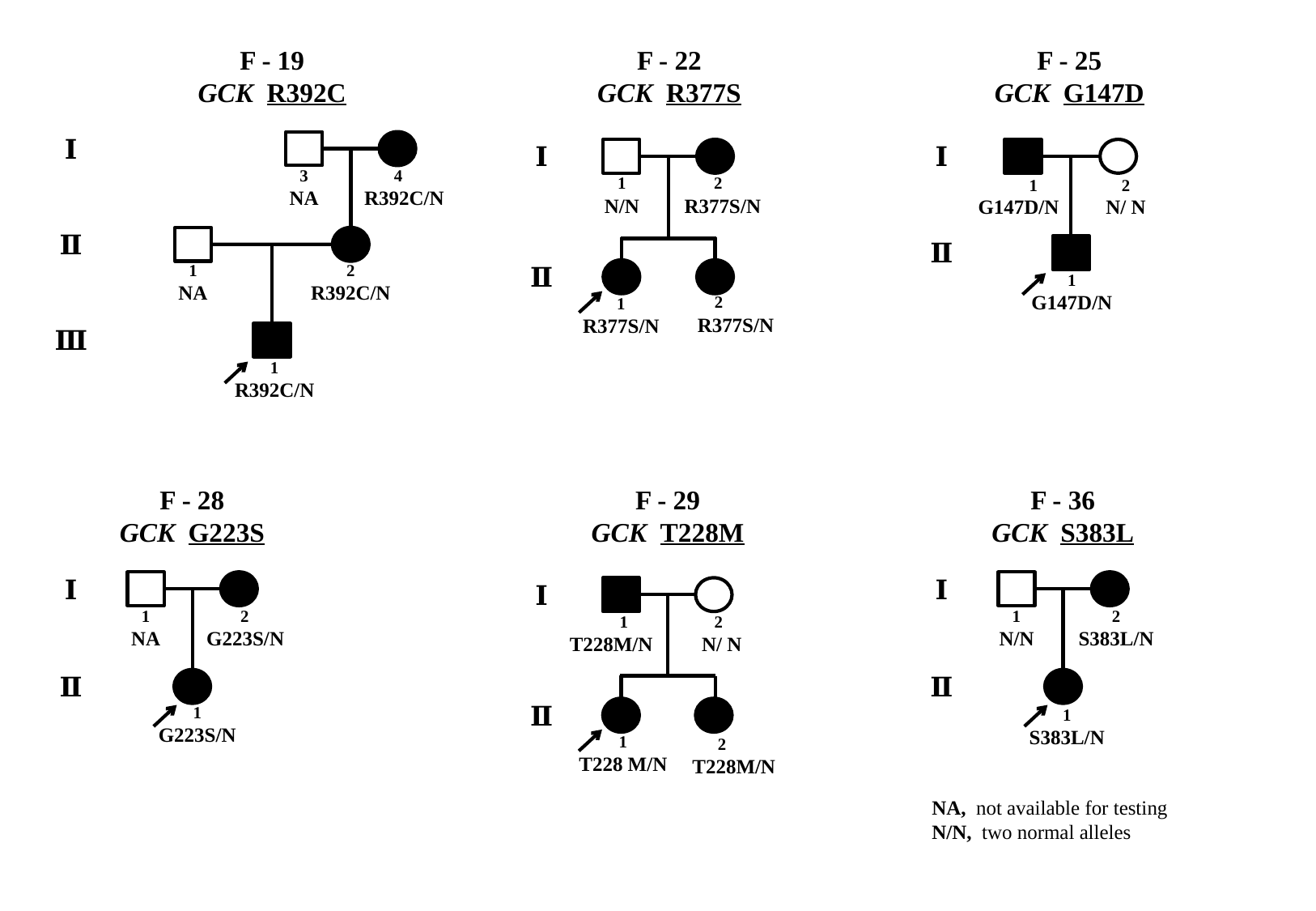

F - 19
GCK R392C
Ⅰ
3
NA
 4
R392C/N
Ⅱ
1
NA
2
R392C/N
Ⅲ
1
R392C/N
F - 22
GCK R377S
Ⅰ
1
N/N
 2
R377S/N
Ⅱ
 2
R377S/N
1
R377S/N
F - 25
GCK G147D
Ⅰ
　 1
G147D/N
2
N/ N
Ⅱ
1
G147D/N
F - 28
GCK G223S
Ⅰ
1
NA
 2
G223S/N
Ⅱ
1
G223S/N
F - 29
GCK T228M
Ⅰ
 1
T228M/N
 2
N/ N
Ⅱ
1
T228 M/N
 2
T228M/N
F - 36
GCK S383L
Ⅰ
1
N/N
2
S383L/N
Ⅱ
1
S383L/N
NA, not available for testing
N/N, two normal alleles

## Slide 4
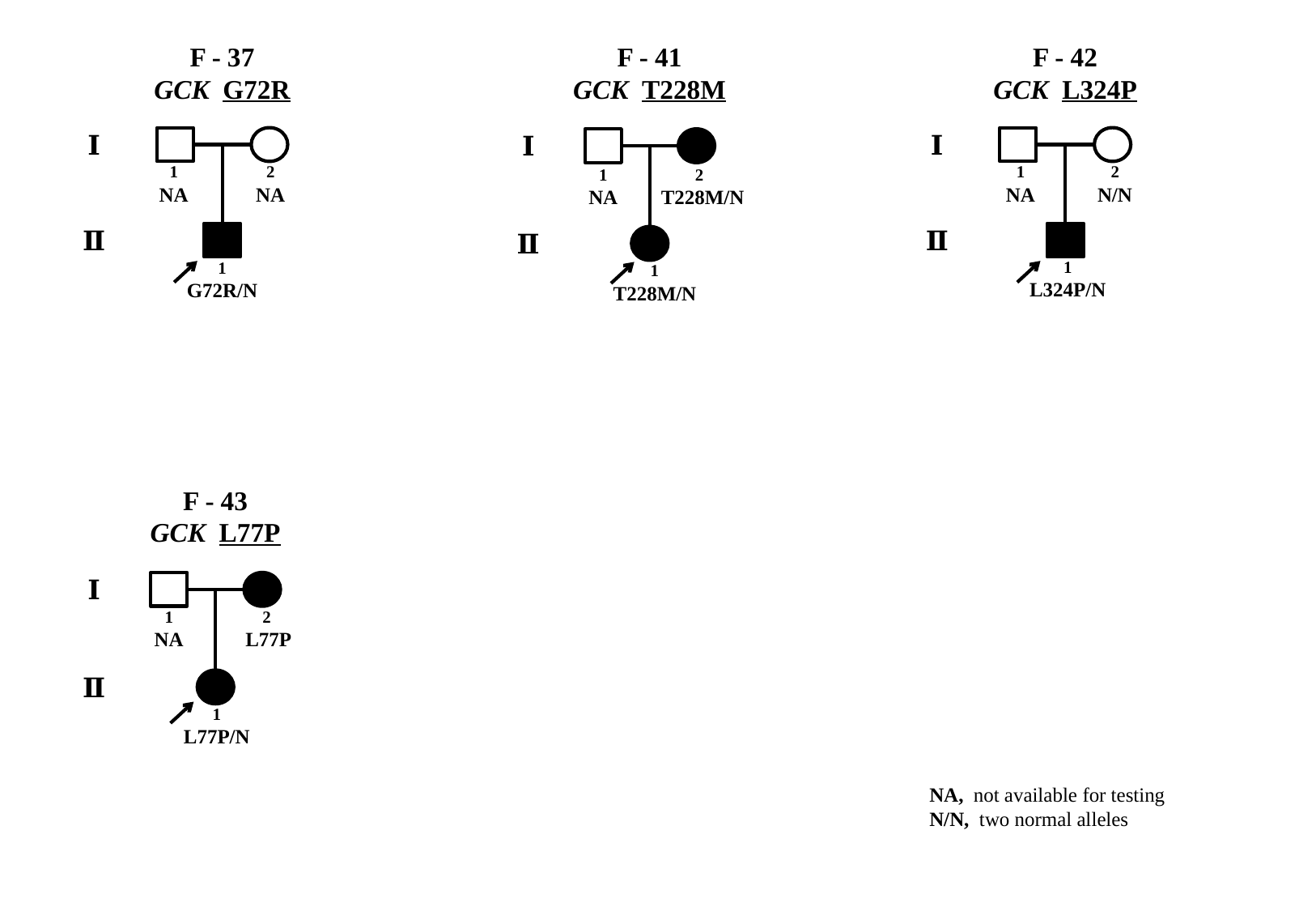

F - 37
GCK G72R
Ⅰ
1
NA
2
NA
Ⅱ
1
G72R/N
F - 41
GCK T228M
Ⅰ
1
NA
 2
T228M/N
Ⅱ
1
T228M/N
F - 42
GCK L324P
Ⅰ
1
NA
2
N/N
Ⅱ
1
L324P/N
F - 43
GCK L77P
1
NA
 2
L77P
1
L77P/N
Ⅰ
Ⅱ
NA, not available for testing
N/N, two normal alleles

## Slide 5
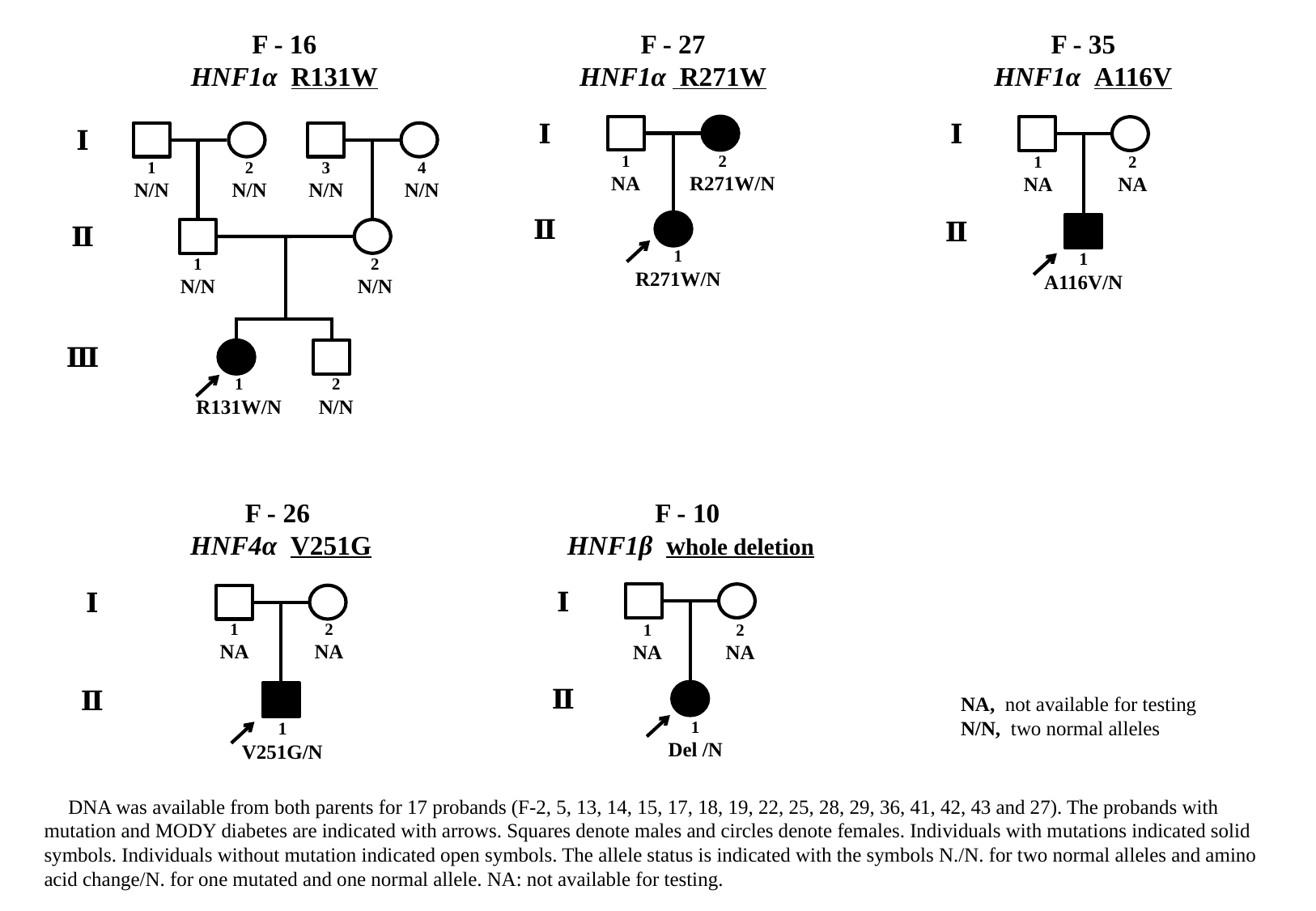

F - 16
HNF1α R131W
Ⅰ
1
N/N
2
N/N
3
N/N
4
N/N
Ⅱ
1
N/N
2
N/N
Ⅲ
1
R131W/N
2
N/N
F - 27
HNF1α R271W
1
NA
 2
 R271W/N
1
R271W/N
F - 35
HNF1α A116V
Ⅰ
1
NA
2
NA
Ⅱ
1
A116V/N
Ⅰ
Ⅱ
F - 26
HNF4α V251G
Ⅰ
1
NA
2
NA
Ⅱ
1
V251G/N
F - 10
HNF1β whole deletion
Ⅰ
1
NA
2
NA
Ⅱ
1
Del /N
NA, not available for testing
N/N, two normal alleles
DNA was available from both parents for 17 probands (F-2, 5, 13, 14, 15, 17, 18, 19, 22, 25, 28, 29, 36, 41, 42, 43 and 27). The probands with mutation and MODY diabetes are indicated with arrows. Squares denote males and circles denote females. Individuals with mutations indicated solid symbols. Individuals without mutation indicated open symbols. The allele status is indicated with the symbols N./N. for two normal alleles and amino acid change/N. for one mutated and one normal allele. NA: not available for testing.
