# Supplementary material for: Identification of Novel GCK and HNF4α Gene Variants in Japanese Pediatric Patients with Onset of Diabetes before 17 Years of Age
Source: J Diabetes Res. 2021 Oct 29;2021:7216339. doi: 10.1155/2021/7216339 (PMC8570896; doi:10.1155/2021/7216339)
Supplement: Supplementary Materials — Supplemental Table 1: primers used for HNF4α, GCK, HNF1α, and HNF1β genes for sequencing analysis. Supplemental Table 2: clinical characteristics of MODY cases with or without variants in each gene. Supplemental Figure 1: sequences of two novel GCK gene mutations and one novel HNF4α gene mutation. Supplemental Figure 2: results of array CGH (human genome CGH array 244K). Supplemental Figure 3: pedigrees of the families of the probands with mutations in GCK, HNF1α, HNF4α, and HNF1β genes, respectively. [file 7216339.f1.zip › Supplementall Figure 2.pptx]

## Slide 1
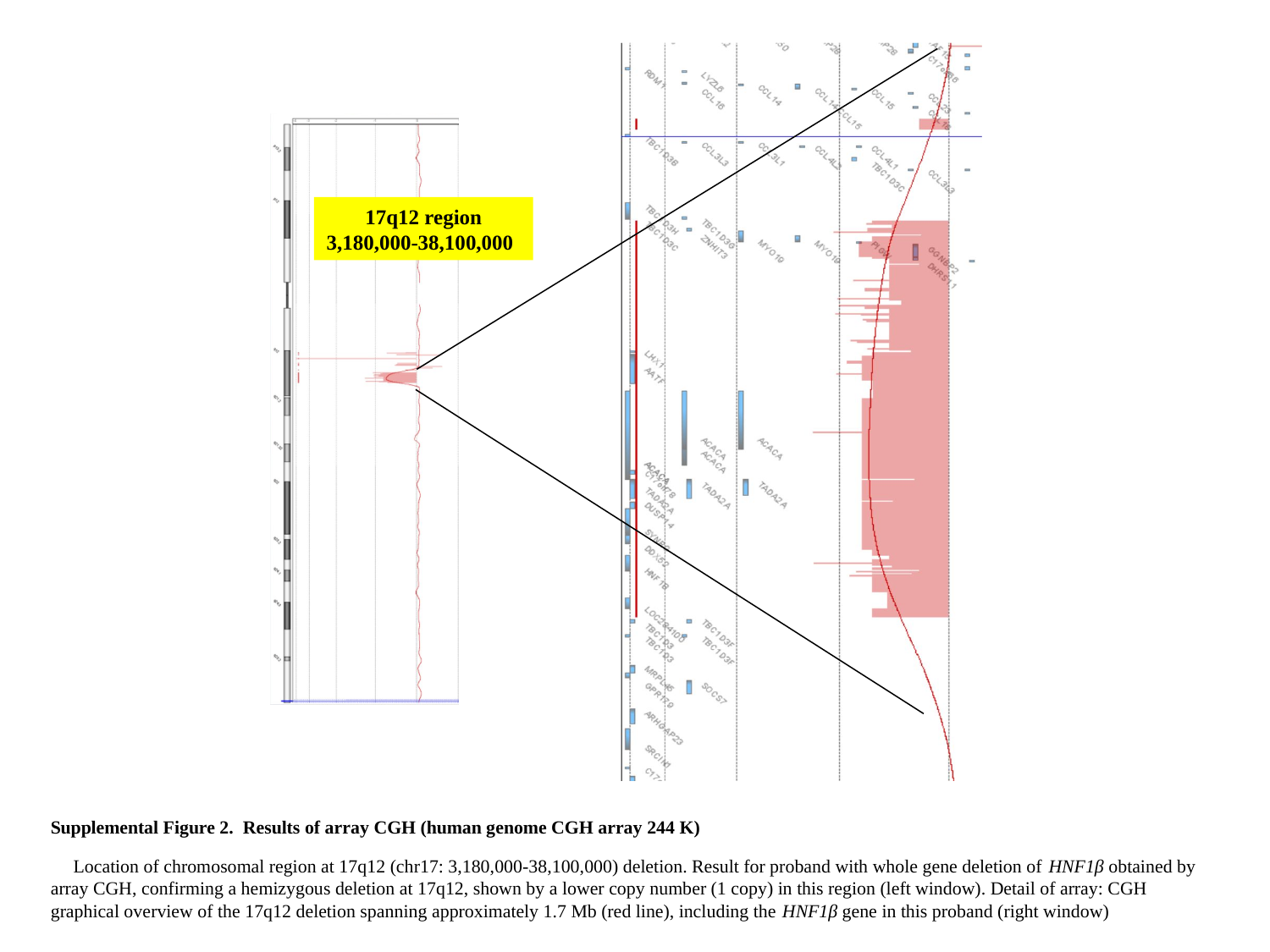

17q12 region
3,180,000-38,100,000
Supplemental Figure 2. Results of array CGH (human genome CGH array 244 K)
　Location of chromosomal region at 17q12 (chr17: 3,180,000-38,100,000) deletion. Result for proband with whole gene deletion of HNF1β obtained by array CGH, confirming a hemizygous deletion at 17q12, shown by a lower copy number (1 copy) in this region (left window). Detail of array: CGH graphical overview of the 17q12 deletion spanning approximately 1.7 Mb (red line), including the HNF1β gene in this proband (right window)
