# Supplementary material for: Identification of Novel GCK and HNF4α Gene Variants in Japanese Pediatric Patients with Onset of Diabetes before 17 Years of Age
Source: J Diabetes Res. 2021 Oct 29;2021:7216339. doi: 10.1155/2021/7216339 (PMC8570896; doi:10.1155/2021/7216339)
Supplement: Supplementary Materials — Supplemental Table 1: primers used for HNF4α, GCK, HNF1α, and HNF1β genes for sequencing analysis. Supplemental Table 2: clinical characteristics of MODY cases with or without variants in each gene. Supplemental Figure 1: sequences of two novel GCK gene mutations and one novel HNF4α gene mutation. Supplemental Figure 2: results of array CGH (human genome CGH array 244K). Supplemental Figure 3: pedigrees of the families of the probands with mutations in GCK, HNF1α, HNF4α, and HNF1β genes, respectively. [file 7216339.f1.zip › Supplemental Figure 1.doc]

A)	B)

　　　

**C)**

Supplemental Figure 1.　Sequences of two novel *GCK* gene and one novel *HNF4α* gene mutations

A) The mutations are sequenced as G147D (c.440 G>A, p. Gly 147 Asp) in *GCK* gene.

B) The mutations are sequenced as N254D (c.760 A>G, p. Asn 254 Asp) in *GCK* gene.

C) The mutations are sequenced as V251G (c.752 T>G, p. Val 251 Gly) in *HNF4α* gene.

The red arrows indicate the positions of the mutations.
