# Supplementary material for: Identification of Novel GCK and HNF4α Gene Variants in Japanese Pediatric Patients with Onset of Diabetes before 17 Years of Age
Source: J Diabetes Res. 2021 Oct 29;2021:7216339. doi: 10.1155/2021/7216339 (PMC8570896; doi:10.1155/2021/7216339)
Supplement: Supplementary Materials — Supplemental Table 1: primers used for HNF4α, GCK, HNF1α, and HNF1β genes for sequencing analysis. Supplemental Table 2: clinical characteristics of MODY cases with or without variants in each gene. Supplemental Figure 1: sequences of two novel GCK gene mutations and one novel HNF4α gene mutation. Supplemental Figure 2: results of array CGH (human genome CGH array 244K). Supplemental Figure 3: pedigrees of the families of the probands with mutations in GCK, HNF1α, HNF4α, and HNF1β genes, respectively. [file 7216339.f1.zip › Supplemental Table 1.doc]

Supplemental Table 1. Primers used for *HNF4α*, *GCK*, *HNF1α*, and *HNF1β* genes for sequencing analysis

| Gene symbol | Region |  | Primer sequence | Location | Amplicon size (bp) | Annealing temperature |
| --- | --- | --- | --- | --- | --- | --- |
|  |  |  |  |  |  |  |
| ***HNF4α*** | Exon 1 | Forward | 5'-GGGGGACCGATTAACCATTA-3' | 5'UTR | 411 | 59 °C |
|  |  | Reverse | 5'-GTTGAGTGCCAAAGATCTGC-3' | intron 1 |  |  |
|  |  |  |  |  |  |  |
|  | Exon 2 | Forward | 5'-CTCCCTTAGATGCCTGACAT-3' | intron 1 | 317 | 58 °C |
|  |  | Reverse | 5'-CCACTCAGGGAGAAGACAGA-3' | intron 2 |  |  |
|  |  |  |  |  |  |  |
|  | Exon 3 | Forward | 5'-GGGATGAAGAGATGAGAGCA-3' | intron 2 | 346 | 58 °C |
|  |  | Reverse | 5'-TTCTCAGCCATTAGCCAGTC-3' | intron 3 |  |  |
|  |  |  |  |  |  |  |
|  | Exon 4 | Forward | 5'-TAGAGGCCATCTCCCCTCA-3' | intron 3 | 409 | 58 °C |
|  |  | Reverse | 5'-ATGTGAAACCGGACTCAGTG-3' | intron 4 |  |  |
|  |  |  |  |  |  |  |
|  | Exon 5 | Forward | 5'-CTCCCCACATCTGATTCCAG-3' | intron 4 | 444 | 58 °C |
|  |  | Reverse | 5'-TTGTTGGAGGAGCTAACTGA-3' | intron 5 |  |  |
|  |  |  |  |  |  |  |
|  | Exon 6 | Forward | 5'-AGCGTCACTGAGTTGGCTA-3' | intron 5 | 304 | 58 °C |
|  |  | Reverse | 5'-TCTAATGCAAGCCTGAGGAC-3' | intron 6 |  |  |
|  |  |  |  |  |  |  |
|  | Exon 7 | Forward | 5'-CACCAGCTATCTTGCCAACT-3' | intron 6 | 297 | 58 °C |
|  |  | Reverse | 5'-GCGTTCTGGAGAGAGAGTC-3' | intron 7 |  |  |
|  |  |  |  |  |  |  |
|  | Exon 8_1 | Forward | 5'-CTTGTGTGATACAAGTCAGG-3' | intron 7 | 460 | 55 °C |
|  |  | Reverse | 5'-GTTAAGCAACTTGCCCAAAG-3' | intron 8 |  |  |
|  |  |  |  |  |  |  |
|  | Exon 9 | Forward | 5'-CCCAGGAATAGGTACCCAA-3' | intron 8 | 357 | 58 °C |
|  |  | Reverse | 5'-TTCTCCTGCCTCCTGTCTTA-3' | intron 9 |  |  |
|  |  |  |  |  |  |  |
|  | Exon 10 | Forward | 5'-CCGGGCCTCTTCATTTACTC-3' | intron 9 | 496 | 58 °C |
|  |  | Reverse | 5'-ATGGATGAAGGTGAAGGTGA-3' | exon 10 |  |  |
|  |  |  |  |  |  |  |
|  | Exon 1  (isoform e) | Forward | 5'-CCACCTTGGGTGATTAGAAG-3' | 5'UTR | 294 | 58 °C |
|  |  | Reverse | 5'-TAAGAAACACACGGGGAGGT-3' | 5'UTR |  |  |
|  |  |  |  |  |  |  |
|  | Exon 8_2  (isoform c, f) | Forward | 5'-AACCTGTTGCAGGAGATGCT-3 | intron 8 | 491 | 58 °C |
|  |  | Reverse | 5'-AGCACAAGAGTCACCACTTC-3 | intron 8 |  |  |
|  |  |  |  |  |  |  |

| Gene symbol | Region |  | Primer sequence | Location | Amplicon size (bp) | Annealing temperature |
| --- | --- | --- | --- | --- | --- | --- |
|  |  |  |  |  |  |  |
| ***GCK*** | Exon 1 | Forward | 5'-TGGTCACCATGACAACCACA-3' | 5'UTR | 701 | 58 °C |
|  |  | Reverse | 5'-CTCAAAGAGCCTGTGCATAA-3' | intron 1 |  |  |
|  |  |  |  |  |  |  |
|  | Exon 2 | Forward | 5'-GTTTGAGAAGATGGGTCATC-3' | intron 1 | 476 | 58 °C |
|  |  | Reverse | 5'-AAGTCGAGGACTTCATTGCT-3' | intron 2 |  |  |
|  |  |  |  |  |  |  |
|  | Exon 3 | Forward | 5'-TCTTTGTAATATCCGGGCTCA-3' | intron 2 | 425 | 58 °C |
|  |  | Reverse | 5'-CATGTTGGCCAGTCCTGTT-3' | intron 3 |  |  |
|  |  |  |  |  |  |  |
|  | Exon 4 | Forward | 5'-TTGCAGTGTCCCTGAGGAAT-3' | intron 3 | 301 | 58 °C |
|  |  | Reverse | 5'-GGGCTACATTTGAAGGCAG-3' | intron 4 |  |  |
|  |  |  |  |  |  |  |
|  | Exon 5+6 | Forward | 5'-TAGCACCCTGCCTCCAGTA-3' | intron 4 | 485 | 58 °C |
|  |  | Reverse | 5'-CAGGCTCTGCTCTGACATC-3' | intron 6 |  |  |
|  |  |  |  |  |  |  |
|  | Exon 7 | Forward | 5'-CCATTGTTCCAGACAAAGCA-3' | intron 6 | 400 | 58 °C |
|  |  | Reverse | 5'-CAAGCCCATTATCTGCAATG-3' | intron 7 |  |  |
|  |  |  |  |  |  |  |
|  | Exon 8 | Forward | 5'-AGGGAAAGACGTGAACCAG-3' | intron 7 | 392 | 58 °C |
|  |  | Reverse | 5'-TCGCCCTGAGACCAAGTCT-3' | intron 8 |  |  |
|  |  |  |  |  |  |  |
|  | Exon 9 | Forward | 5'-TTAGTTAGAGGGGGCAGTA-3' | intron 8 | 562 | 58 °C |
|  |  | Reverse | 5'-CTACGAAATCTTGGAGCTTG-3' | intron 9 |  |  |
|  |  |  |  |  |  |  |
|  | Exon 10 | Forward | 5'-CAAGCTCCAAGATTTCGTAG-3' | intron 9 | 557 | 58 °C |
|  |  | Reverse | 5'-GGAAATTGATTCCAGCGAGA-3' | exon 10 |  |  |
|  |  |  |  |  |  |  |
|  | Exon 1  (isoform 3) | Forward | 5'-TGTCAACTGTTACCCACATG-3' | intron 1 | 405 | 58 °C |
|  |  | Reverse | 5'-CCCAGCCTTAGTTTTGGTAA-3' | intron 1 |  |  |
|  |  |  |  |  |  |  |
|  | Exon 2  (isoform 3) | Forward | 5'-TCTCCCACTGCTATCCAAC-3' | intron 1 | 327 | 58 °C |
|  |  | Reverse | 5'-CAGGCTTGGTGAAAGCGAT-3' | intron 1 |  |  |
|  |  |  |  |  |  |  |

| Gene symbol | Region |  | Primer sequence | Location | Amplicon size (bp) | Annealing temperature |
| --- | --- | --- | --- | --- | --- | --- |
|  |  |  |  |  |  |  |
| ***HNF1α*** | Exon 1 | Forward | 5'-AGGAGTTTGGTTTGTGTCTG-3' | 5'UTR | 502 | 59 °C |
|  |  | Reverse | 5'-GGCTCGTTAGGAGCTGAG-3' | intron 1 |  |  |
|  |  |  |  |  |  |  |
|  | Exon 2 | Forward | 5'-CTCCATAACTGCTTTCATGCA-3' | intron 1 | 393 | 58 °C |
|  |  | Reverse | 5'-ACCTATGAGTTAGGGGAGAG-3' | intron 2 |  |  |
|  |  |  |  |  |  |  |
|  | Exon 3 | Forward | 5'-AAAGAATCAAGGGCAAGGTC-3' | intron 2 | 309 | 58 °C |
|  |  | Reverse | 5'-CAGACCAAACCAGCACTGT-3' | intron 3 |  |  |
|  |  |  |  |  |  |  |
|  | Exon 4 | Forward | 5'-AGAGCTCAGCTTCTCAGAA-3' | intron 3 | 442 | 58 °C |
|  |  | Reverse | 5'-ATGGAACCAAACTGAAGTGC-3' | intron 4 |  |  |
|  |  |  |  |  |  |  |
|  | Exon 5-6 | Forward | 5'-TTGAAGTGCTGAGGGCTGT-3' | intron 4 | 633 | 58 °C |
|  |  | Reverse | 5'-TCTTCCAGCTCCTGGATTC-3' | intron 6 |  |  |
|  |  |  |  |  |  |  |
|  | Exon 7 | Forward | 5'-CAGGGGTGGGATATAACTG-3' | intron 6 | 346 | 58 °C |
|  |  | Reverse | 5'-CATGCAGACTGCAATGCCT-3' | intron 7 |  |  |
|  |  |  |  |  |  |  |
|  | Exon 8+9 | Forward | 5'-CTGGGACTAGGGCTGTCA-3' | intron 7 | 647 | 58 °C |
|  |  | Reverse | 5'-TCCCTGAGATGTTCTTGTAA-3' | intron 9 |  |  |
|  |  |  |  |  |  |  |
|  | Exon 10 | Forward | 5'-AGTACCCCTAGGGACAGG-3' | intron 9 | 447 | 58 °C |
|  |  | Reverse | 5'-CATGAACAGGCTTTGCTCCT-3' | exon 10 |  |  |
|  |  |  |  |  |  |  |

| Gene symbol | Region |  | Primer sequence | Location | Amplicon size (bp) | Annealing temperature |
| --- | --- | --- | --- | --- | --- | --- |
|  |  |  |  |  |  |  |
| ***HNF1β*** | Exon 1 | Forward | 5'-CTTCCCACTAATTTGCATATC-3' | 5'UTR | 689 | 58 °C |
|  |  | Reverse | 5'-GACTTCTCTGGTGGGAAAC-3' | intron 1 |  |  |
|  |  |  |  |  |  |  |
|  | Exon 2 | Forward | 5'-CCCCAGATGTCTCCCACTA-3' | intron 1 | 298 | 58 °C |
|  |  | Reverse | 5'-AGGGCAAAGGTCACTTCAG-3' | intron 2 |  |  |
|  |  |  |  |  |  |  |
|  | Exon 3 | Forward | 5'-TTCGTCCGTTGTCTGTCTGT-3' | intron 2 | 488 | 58 °C |
|  |  | Reverse | 5'-GGTTCTGTGGAACATACTTTG-3' | intron 3 |  |  |
|  |  |  |  |  |  |  |
|  | Exon 4 | Forward | 5'-CAACCAAGACTGCTGTGATT-3' | intron 3 | 435 | 58 °C |
|  |  | Reverse | 5'-TCTCAAAAGCGTTTGCTCCT-3' | intron 4 |  |  |
|  |  |  |  |  |  |  |
|  | Exon 5 | Forward | 5'-CTGGAGAAAAGCAGCCGAT-3' | intron 4 | 392 | 58 °C |
|  |  | Reverse | 5'-CCTTGTGAGAAGTTGTGTTT-3' | intron 5 |  |  |
|  |  |  |  |  |  |  |
|  | Exon 6 | Forward | 5'-GCTCTTTGTGGTCCAAGTC-3' | intron 5 | 294 | 58 °C |
|  |  | Reverse | 5'-TCGTGGGTGAGTTTGAAGG-3' | intron 6 |  |  |
|  |  |  |  |  |  |  |
|  | Exon 7 | Forward | 5'-CCATGGAATCTCCTGTGTAA-3' | intron 6 | 443 | 58 °C |
|  |  | Reverse | 5'-TAGGCAGGGAAAAGTGACCA-3' | intron 7 |  |  |
|  |  |  |  |  |  |  |
|  | Exon 8 | Forward | 5'-AACCACCAGCCCTTTTATCT-3' | intron 7 | 311 | 58 °C |
|  |  | Reverse | 5'-CACAACCTCTGCACATCCAT-3' | intron 8 |  |  |
|  |  |  |  |  |  |  |
|  | Exon 9 | Forward | 5'-TTGGGCATCATCTCCCTTAG-3' | intron 8 | 456 | 58 °C |
|  |  | Reverse | 5'-GTTTTCGCATCAGTTTGTTCG-3' | intron 9 |  |  |
|  |  |  |  |  |  |  |
