# Supplementary material for: Identification of Novel GCK and HNF4α Gene Variants in Japanese Pediatric Patients with Onset of Diabetes before 17 Years of Age
Source: J Diabetes Res. 2021 Oct 29;2021:7216339. doi: 10.1155/2021/7216339 (PMC8570896; doi:10.1155/2021/7216339)
Supplement: Supplementary Materials — Supplemental Table 1: primers used for HNF4α, GCK, HNF1α, and HNF1β genes for sequencing analysis. Supplemental Table 2: clinical characteristics of MODY cases with or without variants in each gene. Supplemental Figure 1: sequences of two novel GCK gene mutations and one novel HNF4α gene mutation. Supplemental Figure 2: results of array CGH (human genome CGH array 244K). Supplemental Figure 3: pedigrees of the families of the probands with mutations in GCK, HNF1α, HNF4α, and HNF1β genes, respectively. [file 7216339.f1.zip › Supplemental Table 2.doc]

Supplemental Table 2. Clinical characteristics of MODY cases with or without variants in each gene

|  | Probands with *HNF4a* variants | Probands with *GCK* variants | Probands with *HNF1α* variants | Probands with *HNF1β* variants | MODY X (no variant) |
| --- | --- | --- | --- | --- | --- |
|  |  |  |  |  |  |
| n (male / female) | 1 (1 / 0) | 24 (12 /12) | 3 (1 /2) | 1 (0 / 1) | 16 (3/ 13) |
| Age at diagnosis of diabetes [year (range)] | 13.0 | 9.9 ± 3.85 | 10.0 ± 3.46 | 14.0 | 11.1 ± 3.57 |
|  | - | (2.1 - 17) | (8 - 14) | - | (5 - 16) |
| BMI (kg/m^2^) | 21.9 | 16.7 ± 2.29 | 17.5 ± 2.30 | 19.3 | 19.6 ± 2.60 |
| HbA1c (%) (NGSP) | 7.8 | 6.6 ± 0.40 | 6.4 ± 0.26 | 8.9 | 9.0 ± 2.00 |
| (mmol/mol) (SI) | 62 | 49 | 46 | 74 | 75 |
| BG concentrations (mg/dl) |  |  |  |  |  |
| OGTT 0 min | 136 | 126.1 ± 10.62 | 98.7 ± 8.50 | 225 | 140.9 ± 43.42 |
| OGTT 60 min | 210 | 199.2 ± 32.38 | 215.7 ± 16.77 | 226 | 278.9 ± 77.73 |
| IRI (μU/ml) |  |  |  |  |  |
| OGTT 0 min | 1.0 | 5.5 ± 2.30 | 3.2 ± 2.30 | ND | 6.1 ± 5.06 |
| OGTT 60 min | 7.4 | 49.8 ± 28.10 | 21.3 ± 6.10 | 20.3 | 22.9 ± 17.23 |
| ^a)^ Family history of diabetes (%) | 1 (100%) | 23 (95.8%) | 3 (100%) | ND | 15 (94%) |
| In one parent | 1 (100%) | 20 (83.3%) | 2 (66.7%) | ND | 15 (94%) |

NGSP, National Glycohemoglobin Standardization Program; SI, International System of Units; ND, no data.

Age at the time of diagnosis, blood glucose concentrations, HbA1c and insulin dose are represented as means ± standard deviation.

^a)^ Number of children with a sibling or first–third-degree relative with diabetes.
